# The hypothalamus for whole-body physiology: from metabolism to aging

**DOI:** 10.1007/s13238-021-00834-x

**Published:** 2021-04-07

**Authors:** Tiemin Liu, Yong Xu, Chun-Xia Yi, Qingchun Tong, Dongsheng Cai

**Affiliations:** 1 State Key Laboratory of Genetic Engineering, Department of Endocrinology and Metabolism, Institute of Metabolism and Integrative Biology, Human Phenome Institute, and Collaborative Innovation Center for Genetics and Development, Zhongshan Hospital, School of Life Sciences, Fudan University, Shanghai, 200438 China; 2 Children’s Nutrition Research Center, Department of Pediatrics, Department of Molecular and Cellular Biology, Baylor College of Medicine, One Baylor Plaza, Houston, TX 77030 USA; 3 Department of Endocrinology and Metabolism, Amsterdam University Medical Centers, Amsterdam Gastroenterology Endocrinology Metabolism, University of Amsterdam, Meibergdreef 9, 1105AZ Amsterdam, Netherlands; 4 Brown Foundation Institute of Molecular Medicine, Department of Neurobiology and Anatomy, University of Texas McGovern Medical School, Graduate Program in Neuroscience of MD Anderson UTHealth Graduate School of Biomedical Sciences, Houston, TX 77030 USA; 5 Department of Molecular Pharmacology, Albert Einstein College of Medicine, Bronx, New York, NY 10461 USA

**Keywords:** neuron, metabolism, hypothalamus, obesity, aging

## Abstract

Obesity and aging are two important epidemic factors for metabolic syndrome and many other health issues, which contribute to devastating diseases such as cardiovascular diseases, stroke and cancers. The brain plays a central role in controlling metabolic physiology in that it integrates information from other metabolic organs, sends regulatory projections and orchestrates the whole-body function. Emerging studies suggest that brain dysfunction in sensing various internal cues or processing external cues may have profound effects on metabolic and other physiological functions. This review highlights brain dysfunction linked to genetic mutations, sex, brain inflammation, microbiota, stress as causes for whole-body pathophysiology, arguing brain dysfunction as a root cause for the epidemic of aging and obesity-related disorders. We also speculate key issues that need to be addressed on how to reveal relevant brain dysfunction that underlines the development of these disorders and diseases in order to develop new treatment strategies against these health problems.

## Introduction

With the development of life quality, people eat better and live longer. Obesity and population aging have now become a great burden to people in modern society. According to the World Health Organization, more than 650 million adults worldwide are obese; till 2050, two billion people would be older than 65 years of age (Organization, 2015). Given the increasing prevalence of obesity and aging-associated dysfunctions in humans, it is of most importance to understand the mechanism underlying them.

Generally, metabolic dysfunction results from both intrinsic factors and environmental changes. Whole-genome sequencing and genome-wide association studies (GWAS) helped identify many susceptible loci associated with human obesity (Locke et al., 2015); the sexual dimorphism in metabolic regulation has long been emphasized in clinic research (Palmer and Clegg, 2015). As to environmental changes, gut-microbiota imbalance, chronic stress and micro-environmental inflammation have been associated with the obesity pandemic (Flier, 2004; Sominsky and Spencer, 2014; Razzoli et al., 2017; Tomiyama, 2018; Cani et al., 2019).

The brain plays a significant role in energy balance. As early as in 1849, Claude Bernard have observed that stimulation of the base of the fourth ventricle in rabbits caused a dramatic rise in blood glucose (Ruud et al., 2017). However, it was not until recent years that people began to discover neurobiological mechanisms in metabolic regulation with the help of powerful scientific tools. Now people have discovered multiple neural pathways participating in different aspects of metabolism. In addition, recent studies found that not only the key metabolic regulatory neurons, but also the previously largely neglected “supporting cells”, in particularly the microglia and astrocytes surrounding the neurons, are important players in the hypothalamic metabolic regulatory machinery (Thaler et al., 2012; Gao et al., 2014; García-Cáceres et al., 2016, 2019; Vicente-Gutierrez et al., 2019).

Metabolism and aging process influence each other. During aging, there is huge changes in body metabolism, in which the hypothalamus plays an important role. The food-regulatory neurons in the arcuate nucleus of hypothalamus (ARC), micro-inflammation and hypothalamic stem cells all influence aging process, indicating that the brain regulation, especially the hypothalamus, not only plays a significant role in metabolic pathology, but also in other metabolism-related physiology, such as aging.

In this review, we will list some of the most common factors that influence the regulatory functions of the brain on the whole-body metabolism, including gene mutations, sex, brain glial cells, gut microbiota and stress, and dissect molecular mechanisms and related neuronal circuits by which these factors interact with brain to regulate metabolism.

## Genetic Basis for Human Obesity

Recent studies revealed genetic and epigenetic basis for variations in human body mass index (BMI) (Farooqi and O'Rahilly, 2006; Locke et al., 2015; Wahl et al., 2017), and strikingly the majority of BMI-associated genetic variants affect genes that are enriched in the brain (Locke et al., 2015; Turcot et al., 2018). While the roles of many of these genetic variants have not been illustrated yet, here we discussed some genes whose mutations are known to cause human obesity via dysregulations in the central nervous system (CNS) (Table 1).

**Table 1:** Genes that function in the brain and regulate whole-body metabolism.

**Physiological aspect involvements**	**Gene name**	**Mainly locations in the hypothalamus**	**Symptoms in human mutation**	**Phenotypes in mice mutation**
**Leptin-melanocortin pathway**	*LEPR*	ARC, PVH, VMH and LHA	Early-onset obesity	Early-onset obesity
	*POMC*	ARC	Obesity and hyperphagia	Obesity and hyperphagia
	*NCOA1*	Widely expressed	Obesity	Hyperphagia, increased fat mass and diet-induced obesity
	*MC4R*	PVH	Obesity	Obesity, hyperphagia and hyperglucemia
	*SIM1*	PVH	Obesity	Obesity and hyperphagia
	*ARNT2*	Widely expressed	Associated with BMI	Hyperphagia, abnormal glucose metabolism and excessive lipid storage
	*BDNF*	PVH and VMH	Associated with BMI	Disrupted body weight and food intake
	*NTRK2*	ARC, VMH and DMH	Obesity	Obesity and hyperphagia
**Primary cilia related**	*CEP19*	Widely expressed	Obesity	Obesity and hyperphagia
	*ADCY3*	ARC, PVH and VMH	Associated with BMI	Disrupted body weight
**Other genes**	*FTO*	Widely expressed	Associated with BMI and more food intake	Reduced body fat mass (knockout)
	*MYT1L*	Widely expressed	Obesity	/
	*SH2B1*	Widely expressed	Obesity	Obesity and leptin resistance

ADCY3, adenylate cyclase 3; ARC, arcuate nucleus of hypothalamus; ARNT2, Aryl hydrocarbon receptor nuclear translocator 2; BDNF, brain-derived neurotropic factor; BMI, body mass index; CEP19, centrosomal protein 19; DMH, dorsomedial hypothalamus; FTO, FTO alpha-ketoglutarate dependent dioxygenase in *Homo sapiens* and fat mass and obesity associated in *Mus musculus*; LEPR, leptin receptor; LHA, lateral hypothalamic area; MC4R, melanocortin 4 receptor; MYT1L, myelin transcription factor 1 like; NCOA1, nuclear receptor coactivator 1; NTRK2, neurotrophic receptor tyrosine kinase 2; POMC, pro-opiomelanocortin; PVH, paraventricular nucleus of the hypothalamus; SH2B1, SH2B adaptor protein 1; SIM1, single-minded 1; VMH, ventromedial hypothalamus

Many obesity-related genes are involved in the regulation of leptin-melanocortin pathway. The leptin receptor (LEPR) mainly locates in the ARC (Nasrallah and Horvath, 2014). Loss of function mutation in the *LEPR* gene cause early-onset obesity in both humans (Clement et al., 1998) and mice (Cohen et al., 2001). Pro-opiomelanocortin (POMC) neurons are one of neuron types expressing the LEPR. Patients with *POMC* deficiency (Krude et al., 1998) or *Pomc* knockout mice (Yaswen et al., 1999; Shen et al., 2017) have severe metabolic disorders such as obesity and hyperphagia. Actually, not only homozygous *POMC* mutation, but also heterozygous point mutations, which lead to disrupted alpha-melanocyte-stimulating hormone/beta-melanocyte-stimulating hormone (α-MSH/β-MSH, products of the *POMC* gene), could also cause obesity (Farooqi, 2008). Steroid receptor co-activator-1 (SRC-1, encoded by nuclear receptor coactivator 1 gene, *NCOA1*) can enhance *POMC* expression by interacting with phosphorylated signal transducer and activator of transcription 3 (STAT3). When mutating *Ncoa1* in POMC neurons in mice, *Pomc* expression decreased, which led to hyperphagia, increased fat mass and diet-induced obesity. To be noted, *NCOA1* mutated variants are found in obese people with disrupted SRC-1 function, further revealing the functional role of SRC-1 in energy homeostasis (Yang et al., 2019). One of the downstream targets of α-MSH/β-MSH is the melanocortin 4 receptor (MC4R). MC4R mutations are closely associated with obesity in humans (Yeo et al., 1998), and about 1%–2.5% people with severe obesity contain a pathogenic *MC4R* mutation (Farooqi and O'Rahilly, 2006). MC4R inactivation in mice also caused obesity, hyperphagia and hyperglucemia (Huszar et al., 1997), suggesting that *MC4R* is one of the most common obesity-related genes.

Single-minded 1 (SIM1) is abundantly expressed in the paraventricular nucleus of the hypothalamus (PVH). *SIM1* mutations cause of monogenic obesity (Holder et al., 2000), and 13 *SIM1* gene variants are found in obese patients (Ramachandrappa et al., 2013). Studies in mice further confirmed that knocking out *Sim1* gene induced obesity and hyperphagia, probably through melanocortin receptor (Tolson et al., 2010). Aryl hydrocarbon receptor nuclear translocator 2 (ARNT2) is a transcriptional factor which can form heterodimeric complex with SIM1. *Arnt2* mutation in mice with either N-ethyl-N-nitrosourea (ENU) treatment or clustered regularly interspaced short palindromic repeats/Cas9 (CRISPR/Cas9) gene editing causes hyperphagia, abnormal glucose metabolism and excessive lipid storage (Turer et al., 2018). In humans, a research found that one common nsSNP (nonsynonymous single-nucleotide polymorphisms) Gly679Ser/rs4072568 in *ARNT2* was closely associated with BMI and body fat percentage (Swarbrick et al., 2011). In 2013, six children were reported to have homozygous *ARNT2* mutation. In addition to phenotypes such as hypopituitarism, microcephaly and central diabetes insipidus, some, but not all patients showed obese growth curve (Webb et al., 2013). Brain-derived neurotropic factor (BDNF) is initially known to promote neuron development and differentiation by binding to its receptor tropomyosin-related kinase B (TrkB, encoded by the neurotrophic receptor tyrosine kinase 2 gene, *NTRK2*) (Snider, 1994). Human *BDNF* gene polymorphism is proven closely associated with BMI (Hong et al., 2012), and *NTRK2* mutation is also found in obese patients (Yeo et al., 2004). *Bdnf* is highly expressed in the ventromedial nucleus of hypothalamus (VMH) and PVH, regulating body weight and food intake downstream of MC4R signaling in mice (Xu et al., 2003). As to *Ntrk2*, a recent study revealed that *Ntrk2* deletion in the dorsomedial hypothalamus (DMH) of mice caused obesity and hyperphagia (Liao et al., 2019).

In addition to the leptin-melanocortin pathway, some genes that normally build up basic cellular function are recently found to contribute to obesity-related phenotypes. Primary cilia locate on surface of multiple cell types, including neurons. Primary cilia have long been seen as cellular signaling transporter (Siljee et al., 2018). However, in recent years, it has been reported that primary cilia also involve in energy homeostasis. For example, ciliary protein centrosomal protein 19 (CEP19) was found mutated in people with obese symptom, and *Cep19* knockout mice exhibited obesity, hyperphagia and other metabolic abnormalities, similar to human phenotypes (Shalata et al., 2013). In addition, GWAS of BMI speculated that single nucleotide polymorphism (SNP) in adenylate cyclase 3 (*ADCY3*) was strongly related to BMI (Stergiakouli et al., 2014). Indeed, *Adcy3* gene mutated mice became fatter with immobile lifestyle and hyperphagia (Wang et al., 2009). More interestingly, it was reported that MC4R and ADCY3 co-localize in primary cilia of some neurons. When silencing ADCY3 in MC4R-expressing neurons, the regulation of body weight would be disrupted (Siljee et al., 2018). Whether ADCY3 participates in malenocortin pathway has not been clearly illustrated, but current results still pointed out that ADCY3 and other ciliary proteins may profoundly function in whole-body metabolism.

FTO alpha-ketoglutarate dependent dioxygenase (*FTO*, which is called fat mass and obesity associated in *Mus musculus*) gene was firstly identified for development-regulatory function. It was soon found that *FTO* SNPs are associated with BMI and link to more food intake in human. However, in the mouse model, knocking out the *Fto* gene in rodents reduced body fat mass, while overexpression of *Fto* cause obesity and increase of food intake, which is opposite to human studies (Speakman, 2015). Myelin transcription factor 1 like (*MYT1L*) only expresses in human brain. Patients with *MYT1L* single nucleotide variants (SNVs) suffer from intellectual disability and obesity (Loid et al., 2018; Al Tuwaijri and Alfadhel, 2019). Deletion of chromosome 16p11.2, which SH2B adaptor protein 1 (*SH2B1*) locates nearby, causes morbid obesity (Walters et al., 2010). *Sh2b1* knocking out mice had disrupted metabolic phenotypes including obesity and leptin resistance, while restoring SH2B1β in neurons attenuates these metabolic disorders by improving leptin signaling pathways (Ren et al., 2007).

Although the evidence is compelling that the mutation of these genes causes obesity in both humans and rodents, obesity caused by these mutations is rare, which is in contrast to the ever-growing obesity epidemic. Thus, the major cause of the current human obesity is not driven by single gene mutations, but rather by a combination of environmental changes and overall genetic susceptibility.

## Sex Differences in Metabolic Regulation

Sexual differences exist in the regulation of feeding behavior and energy homeostasis in rodents (Shi et al., 2009; Sugiyama and Agellon, 2012). For example, male rats consume higher energy than females even when corrected by their larger lean body mass and metabolic rate (Woodward and Emery, 1989). Further, male rodents are more susceptible to obesity and insulin resistance induced by high fat diet (HFD) feeding compared to female counterparts (Grove et al., 2010; Benz et al., 2012; Stubbins et al., 2012; Yang et al., 2014; Morselli et al., 2016; Dorfman et al., 2017). To understand biological mechanisms for these sex differences, much of effort has been focused on roles of sex chromosomes and gonadal hormones. Emerging evidence also indicates that other factors (encoded by autosome genes) also contribute to sexual dimorphism in energy balance (see Wang and Xu, 2019; Mauvais-Jarvis et al., 2020; Tramunt et al., 2020 for detailed reviews). Here we highlight a few such factors and signals.

### Sex chromosomes

The biological sex is ultimately determined by the sex chromosomes. The sex determining region Y (*Sry*) gene in the Y chromosome in male animals initiates the differentiation of the testes, whereas the absence of the *Sry* gene in females will allow ovaries to develop (Goodfellow and Lovell-Badge, 1993). Using a “four core genotypes’’ mouse model to distinguish the effects of gonadal sex (testes or ovaries) and sex chromosomes (XX or XY) (De Vries et al., 2002), it has been demonstrated that both the gonads (or gonadal hormones) and sex chromosome independently contribute to the sex differences in body weight (Chen et al., 2012; Reue, 2017). A few X-linked genes have been implicated in the regulation of energy balance. One such example is 5-hydroxytryptamine (5-HT) 2C receptor (5-TH_2C_R), encoded by an X-linked gene (*Ht2cr*) (Tecott et al., 1995). Male mice with global deficiency of 5-TH_2C_R develop a late onset hyperphagic obesity (Tecott et al., 1995; Nonogaki et al., 1998). 5-HT and its analogs, e.g., d-fenfluramine and lorcaserin, suppress food intake largely through acting upon the 5-TH_2C_R expressed by POMC neurons (Xu et al., 2008; Berglund et al., 2013; D'Agostino et al., 2018) and dopamine neurons in male animals (Xu et al., 2017). Mechanisms for 5-TH_2C_R’s effects on appetite control involve its actions to activate the transient receptor potential channel 5 (TrpC5) which leads to depolarization of neurons (Gao et al., 2017c). Interestingly, TrpC5 is also encoded by an X-linked gene, and it has been shown to mediate actions of multiple hormones, including estrogen (Qiu et al., 2018), leptin (Qiu et al., 2010; Gao et al., 2017c) and insulin (Qiu et al., 2014). Importantly, deletion of *TrpC5* in POMC neurons leads to obesity in male mice, which is associated with increased daylight feeding and decreased energy expenditure (Gao et al., 2017c). Another X-linked gene is O-GlcNAc transferase (*OGT*), although its functions on body weight balance appears to be site specific, with OGT in orexigenic agouti-related peptide (AgRP) neurons promoting weight gain (Ruan et al., 2014) while OGT in anorexigenic PVH neurons prevent overeating and obesity (Lagerlof et al., 2016). In summary, the *Sry* gene on the Y chromosome determines the development of male or female gonads, which influence the circulating gonadal hormones and therefore energy homeostasis (See below). The X-linked genes also influence the energy balance, but their contributions to the sex difference in body weight control remain to be further investigated.

### Gonadal hormones

#### Estrogen

Estrogen is well known to play an essential role in preventing body weight gain in females, as the withdrawal of endogenous estrogen by ovariectomy (OVX) in female animals leads to body weight gain and hyperadiposity which can be prevented by estrogen treatment but not by progesterone (Drewett, 1973; Schwartz and Wade, 1981; Geary et al., 2001; Wallen et al., 2001; Rogers et al., 2009; Paladini and Roeper, 2014). The most studied estrogen receptor in the context of body weight balance is ERα. Humans or mice with mutations in the ERα (which is also called estrogen receptor 1, *ESR1*) gene are obese (Heine et al., 2000; Okura et al., 2003), and loss of ERα in mice blocks the anorexigenic effects of estrogen (Geary et al., 2001). Brain ERα has been implicated in body weight control (Palmer and Gray, 1986; Butera and Beikirch, 1989; Santollo et al., 2011). Consistently, female mice with *Esr1* deletion in the brain develop obesity (Xu et al., 2011). In particular, ERα is expressed by some POMC neurons in the ARC (Miller et al., 1995; Attia, 2010; de Souza et al., 2011), which can be activated by estrogen (Gao et al., 2007; Malyala et al., 2008; Saito et al., 2015). Female (but not male) mice lacking ERα only in POMC neurons develop hyperphagia and modest body weight gain (Xu et al., 2011). Another hypothalamic ERα population with sexually dimorphic functions is that in the VMH, and multiple groups reported that ERα signals in the VMH in females (but not in males) prevent obesity primarily by stimulating energy expenditure (Musatov et al., 2007; Xu et al., 2011; Martinez de Morentin et al., 2014; Correa et al., 2015). Notably, a recent study used single-cell RNA sequencing to identify a sub-population of VMH neurons co-expressing ERα and reprimo (RPRM), which is only present in female mice and regulate thermogenesis (van Veen et al., 2019). ERα is also abundant in the hindbrain, including the nucleus of the solitary tract (NTS) and dorsal raphe nucleus (DRN) (Osterlund et al., 1998; Merchenthaler et al., 2004; Schlenker and Hansen, 2006), and estrogenic actions in these regions promote satiation (Geary et al., 2001; Asarian and Geary, 2007) and inhibit binge-like eating (Robichaud and Debonnel, 2005; Dalmasso et al., 2011; Santollo et al., 2011; Cao et al., 2014).

It is important to point out that ERα also prevents obesity in males. For example, ERα gene deficiency results in obesity in male mice (Heine et al., 2000; Callewaert et al., 2009) and in men (Smith et al., 1994; Grumbach and Auchus, 1999). Further, male mice lacking ERα in the brain develop modest obesity (Xu et al., 2011). In addition, administration of estrogen or its analogs reduces body weight in male mice (Gao et al., 2007; Finan et al., 2012). Notably, the male gonadal hormone, testosterone, can be converted to estrogen by aromatase (Jarvie and Hentges, 2012) and abundant aromatase is expressed in a few brain regions (Wu et al., 2009). Thus, it is possible that certain male brain regions with high aromatase activity could be exposed to high levels of estrogen despite the lack of circulating estrogen. Indeed, both male and female aromatase knockout mice develop obesity (Jones et al., 2000), highlighting the physiological function of aromatase and estrogen in both sexes. In particular, loss of ERα in the medial amygdala (MeA), a brain region with high aromatase expression (Wu et al., 2009), not only causes obesity in female mice, but also in male mice (Xu et al., 2015).

Collectively, high levels of circulating estrogen prevent obesity in female animals. Many of these estrogenic actions are mediated by multiple ERα populations, which therefore at least partly account for sex differences in body weight. However, ERα in certain brain regions with enriched aromatase activity, e.g., the MeA, also contributes to the maintenance of male energy balance. Notably, two other estrogen receptors, estrogen receptor beta (ERβ) and G protein-coupled receptor 30 (GPR30), are also implicated in the regulation of regulate body weight balance (Foryst-Ludwig et al., 2008; Haas et al., 2009; Sharma et al., 2013; Davis et al., 2014), but their sex-specific functions remain to be further investigated.

#### Testosterone

Testosterone can be converted to estrogen by aromatase (Jarvie and Hentges, 2012). Thus, testosterone actions can be mediated by either the androgen receptor (AR) or estrogen receptors. Notably, AR is encoded by an X-linked gene. Interestingly, while young *Ar* knockout male mice show decreased body weight, aged mutant male mice have increased body weight and adiposity (Sato et al., 2003; Fan et al., 2005; Lin et al., 2005). Consistently, brain-specific deletion of *Ar* leads to a late onset obesity in male mice (Yu et al., 2013), indicating that brain AR is required to prevent aging-associated obesity in males. Further, Fagman et al. reported that whole-body deletion of AR in female mice on an apolipoprotein E (apoE)-deficient background are prone to diet-induced obesity compared to apolipoprotein E (apoE)-deficient female mice (Fagman et al., 2015). While these results suggest potential anti-obesity actions of AR in females, these effects need to be further confirmed in animals with normal apoE functions. Future, testosterone, when exposed to neonatal animals can change structures and excitability of hypothalamic neurons in both males and females (Matsumoto and Arai, 1980), and cause obesity during the adulthood at least in females (Nohara et al., 2011, 2013). Importantly, neonatal administration of dihydrotestosterone, a non-aromatizable androgen, cannot induce similar obese phenotypes in females (Nohara et al., 2011), suggesting that the programming effects of testosterone may be mediated by estrogen receptors, rather than by AR.

### Other factors

While the majority of the field focus on the functions of sex chromosomal genes and gonadal hormones, autosome genes may exist to regulate the sex differences in energy homeostasis.

First, some factors could regulate levels of gonadal hormones or receptors, and therefore contribute to sex differences in energy balance. For example, kisspeptin (Kiss1) and its receptor, G protein-coupled receptor 54 (GPR54), are key regulators of reproduction (Murphy, 2005), and are also required to maintain normal circulating levels of estrogen in females (Seminara et al., 2003; d'Anglemont de Tassigny et al., 2007). Thus, loss of GPR54 leads to massive obesity in female mice but to a lesser extent in males (Tolson et al., 2014, 2016). Similarly, deficiency in angiotensin II receptor (AT2R) renders female mice more prone to diet-induced obesity, associated with reduced estrogen levels, but the same mutation does not affect male mice (Samuel et al., 2013).

In addition, factors that function downstream of gonadal hormones can also contribute to sexual dimorphism in energy balance. One such example is SRC-1. SRC-1 can interact with ERα upon estrogen stimulation, and is required to mediate estrogenic actions to reduce food intake in female mice (Zhu et al., 2013; Yang et al., 2019). Another example is STAT3, which is required to mediate anorexigenic effects of estrogen (Gao et al., 2007). Loss of STAT3 in POMC neurons leads to modest obesity in female mice but does not affect male mice (Xu et al., 2007).

It is important to point out that some autosome gene-encoded factors may contribute to sex differences independent of gonadal hormones. For example, an autosome-encoded transcription factor, TAp63, is found to drive enhanced POMC mRNAs and neural activity in females compared to male counterparts (Wang et al., 2018), corresponding to lower food intake in female mice (Nohara et al., 2011). Deletion of *Tap63* in POMC neurons does not affect male body weight but renders females more susceptible to diet-induced obesity (Wang et al., 2018). Importantly, loss of TAp63 does not influence anorexigenic effects of estrogen (Wang et al., 2018), suggesting that TAp63 and estrogenic actions are largely independent of each other. Interestingly, Sirtuin 1 (SIRT1), a transcriptional target of TAp63 (Su et al., 2012), plays a sex-specific role in POMC neurons to prevent obesity in females but not in males (Ramadori et al., 2010), similar to effects of TAp63. Microglial cells in the hypothalamus also contribute to the sex differences in body weight control. HFD feeding reduces hypothalamic levels of C-X3-C motif chemokine ligand 1 (CX3CL1, a neuron-released chemokine) and its receptor expressed by microglia, CX3CR1, specifically in male mice but not in female mice (Dorfman et al., 2017). Importantly, *Cx3cr1* deletion in female mice causes obesity, associated with “male-like” hypothalamic microglial accumulation and activation (Dorfman et al., 2017).

**Figure 1:**
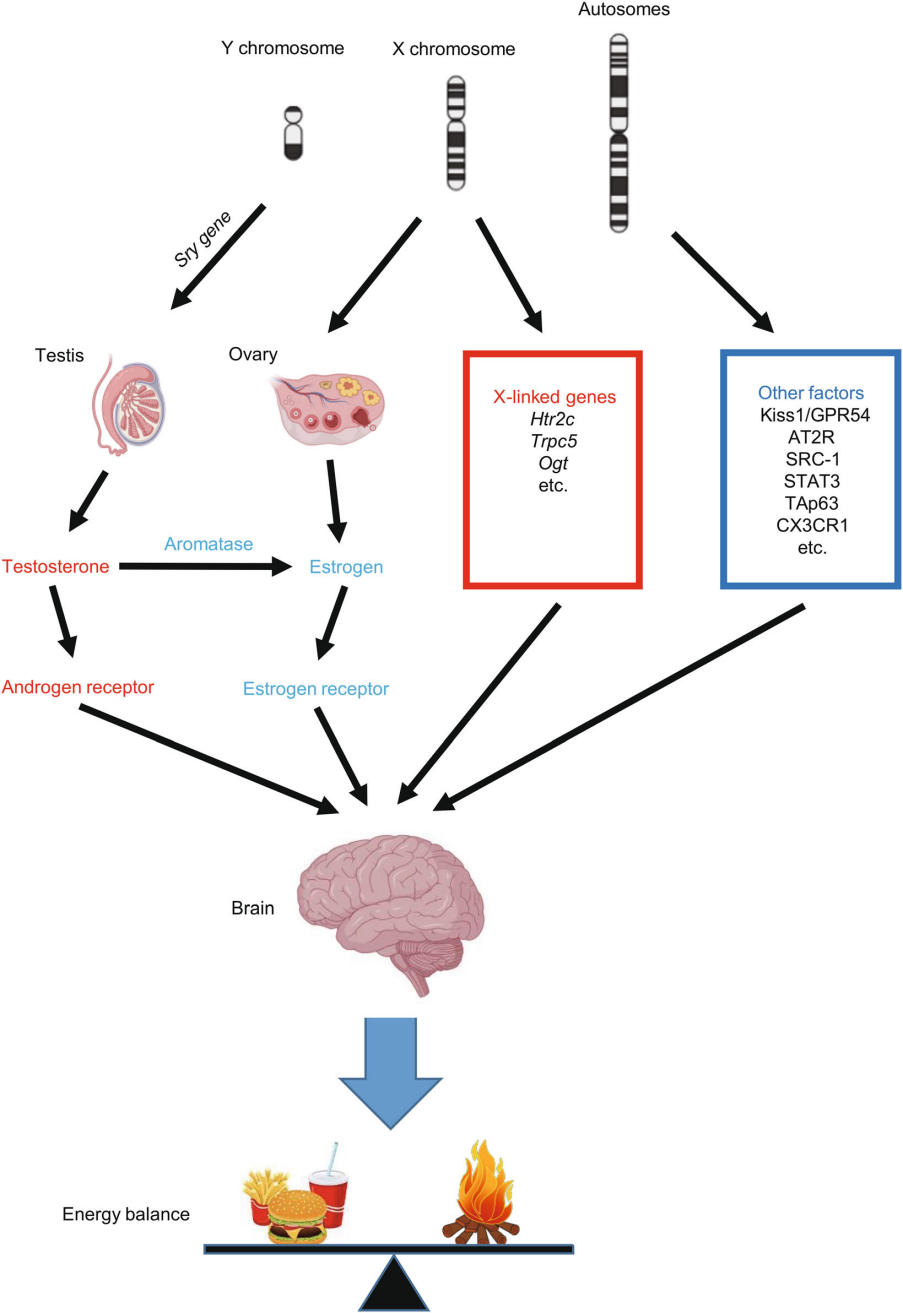
**Mechanisms underlying sex differences in body weight control.** The *Sry* gene in the Y chromosome determines the gonadal development, and therefore influences the circulating levels of estrogen and testosterone. Testosterone can directly act upon the androgen receptor, and can also be converted to estrogen which acts upon the estrogen receptor. These gonadal hormones, together with the X-linked genes and other factors encoded by autosomes, all contribute to sex differences in energy balance partially through their actions in the brain. This figure was illustrated partially via the Biorender.com

In summary, the sex chromosomes are conceivably the fundamental contributors to the sexual dimorphism. The *Sry* gene in the Y chromosome determines the gonadal development, and therefore influence the circulating levels of estrogen and testosterone. Increasing numbers of the X-linked genes are shown to regulate energy balance, although their roles in sex differences remain elusive. Estrogen-ER and testosterone-AR signals in the brain contribute to the regulation of energy balance in females and males, respectively; however, since testosterone can be converted to estrogen in certain brain regions, ER can also partially mediate testosterone’s actions in males. Importantly, autosome-encoded genes also contribute to the sex differences in energy balance through gonadal hormone-dependent or independent mechanisms (Fig. 1).

## Hypothalamic Glial Immune and Metabolic Changes in Obesity and Diabetes

### Microglia innate immunity-driven inflammatory response at frontlines in response to HFD

**Figure 2:**
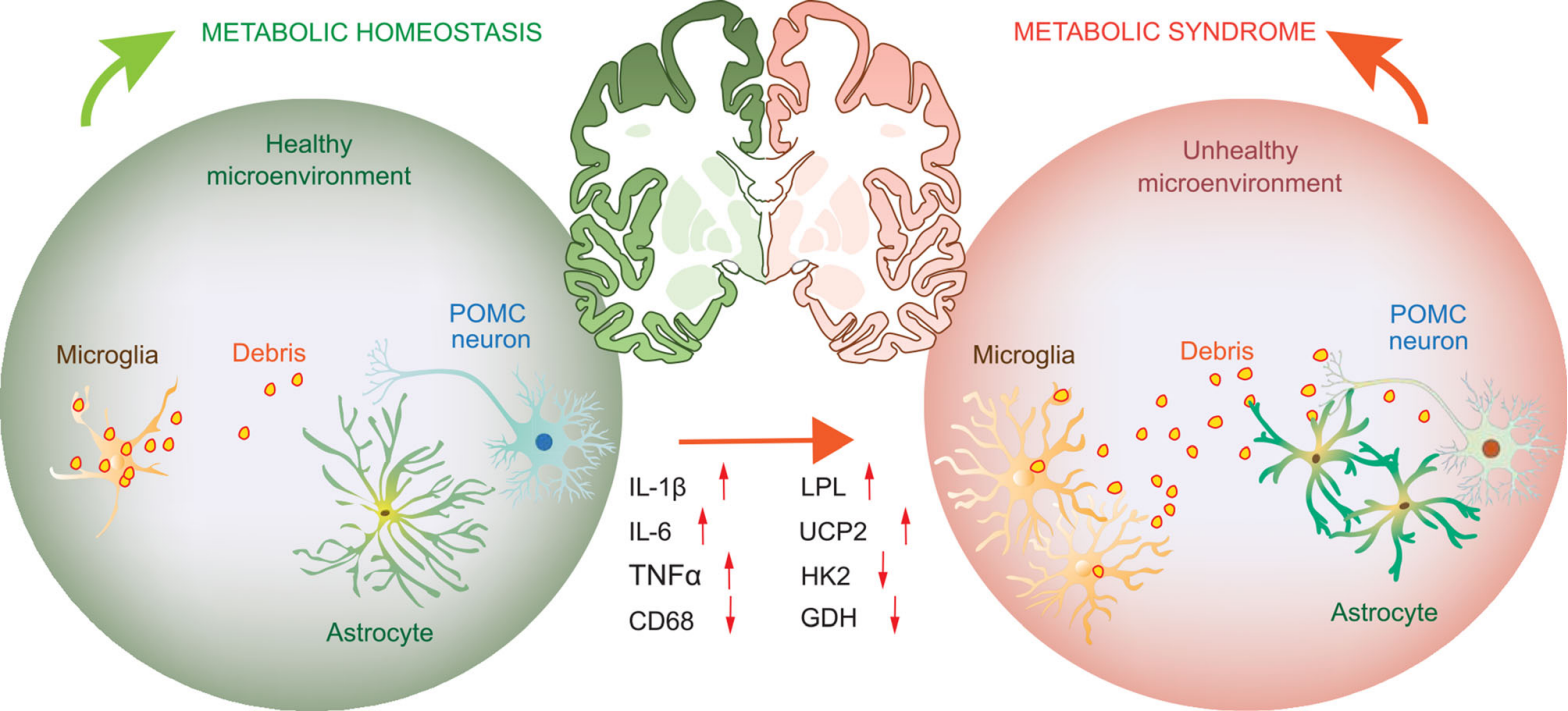
**A schematic illustrates the difference of microglia and astrocytes in MBH between metabolic homeostasis or metabolic syndrome**. In metabolic homeostasis, MBH neurons such as POMC neurons produce regular amount of cell debris and metabolic wastes, matches the phagocytic capacity of the microglia, whereas in metabolic syndrome induced by obesogenic HFD, the over-production of cell debris and wastes drives microglial activation and immunometabolic reprogramming, as evidenced by increased cytokines production (IL-1β, TNFα) and lipids utilization (LPL, UCP2), as well as downregulation of glycolysis and glutamate/glutamine utilization (HK2, GDH). This immunometabolic reprogramming is associated with impaired phagocytic capacity (CD68) in the reactive microglia and increased astrocytes activity, which might contribute to the increased IL-6 production in the local microenvironment. Furthermore, although the number of astrocytes is increased upon HFD, these reactive astrocytes display shortened high-order cell processes, which is an indication of a loss of process plasticity. All these changes in microglia and astrocytes are associated with hypothalamic neuronal dysfunction in controlling food intake and energy homeostasis

Brain microglia are long-surviving and self-renewing innate immune cells, that share the same developmental origin as peripheral tissue macrophages (Ginhoux et al., 2010; Prinz and Priller, 2014; Gomez Perdiguero et al., 2015; Goldmann et al., 2016; Tay et al., 2017). Microglia are crucial for scavenging cell debris and pathogens to maintain brain tissue homeostasis. Various neural disorders in the CNS are associated with microglial dysfunction (Perry et al., 2010; Prinz and Priller, 2014; Heneka et al., 2015). Recent genome-wide analyses showed that microglia have distinctive brain region-dependent transcriptional identities with differences in bioenergetic and immune-regulatory pathways (Grabert et al., 2016). The hypothalamus contains highly heterogeneous and condensed populations of neurons in different regions. The ventromedial arcuate nucleus and median eminence complex at the mediobasal hypothalamus (MBH) is an area lacking normal blood-brain barrier (BBB) (Broadwell et al., 1983; Yi et al., 2006), thus the MBH functions as an important “brain window” for different brain cells sensing blood-born substances (Horvath, 2005; Friedman, 2016; Timper and Bruning, 2017). It is also reasonable that in this region, neural synaptic rewiring events occurs frequently during different metabolic states (Horvath, 2005), and constantly produce cell debris and metabolic waste. In order to keep a healthy and clean microenvironment for the hypothalamic neurons to function, the microglial activity in the MBH needs to match the high demands for immune surveillances and debris phagocytosing/clearances (Fig. 2). This was demonstrated by the fact that microglia in the MBH showed a significantly higher reactivity than in other hypothalamic regions when experimental animals were exposed to HFD, and that this occurred rapidly within 3 days after receiving the HFD (Thaler et al., 2012; Kim et al., 2019). The exact factors that mediate the HFD effects on hypothalamic microglial reactivity is yet unclear. Intriguingly, it is unlikely directly through any major blood-borne components, since microglial cytokines production was not changed upon exposure to the serum derived from HFD-fed animals (Baufeld et al., 2016). Alternatively, the HFD stimulated advanced glycation end-products (AGEs) produced by MBH neurons was proposed to be one of the brain mediators, since AGEs drive microglial inflammatory response via their receptors highly expressed by microglial cells (Gao et al., 2017a), though how the HFD induces AGEs production in the hypothalamic neurons are unknown.

### Immunometabolic reprogramming in proinflammatory microglia

To ensure efficient immune surveillance and phagocytosis, the microglia need to tightly modulate their intracellular metabolic processes to match the fuel demand and control their effector functions. This so-called immunometabolism is one of the key mechanisms for understanding microglial pathophysiology (O'Neill et al., 2016). Unlike the peripheral macrophages that can be defined into a pro-inflammatory (M1) and an anti-inflammatory/pro-resolving (M2) phenotypes, the microglial phenotypes are way more complex following their varied non-macrophage-like functions in the brain (Ransohoff, 2016). It is well-known that M1 macrophages rely mainly on glycolysis, whereas M2 cells are more dependent on oxidative phosphorylation (OXPHOS). However, in HFD-fed animals, the reactive microglia in the MBH likely carry both M1- and M2-like features, since these cells not only express more M1 phenotype-associated pro-inflammatory cytokines such as interleukin 1 beta (IL-1β) and tumor necrosis factor alpha (TNFα) (Thaler et al., 2012), but also more mitochondrial uncoupling protein 2 (UCP2) (Kim et al., 2019), more lipids’ uptake gate-keeper lipoprotein lipase (LPL) (Gao et al., 2017b) and less hexokinase 2 (HK2) (Milanova et al., 2019), indicating an increase of fatty-acid oxidation and OXPHOS and less glycolysis which belong to the M2 phenotype. This illustrates that in HFD-activated microglia, their immunometabolism is reprogrammed differently from the canonical switching process between peripheral M1 and M2 macrophages.

As mentioned above, one important metabolic factor is the mitochondrial UCP2 which is linked to cytokine production in the pro-inflammatory microglia (Cannon and Nedergaard, 2004). At early stage of HFD feeding, the hypothalamic microglia produce more UCP2, which is associated with increases of IL-1β, interleukin-6 (IL-6) and TNFα expression, as well as changes in the mitochondrial number and size (Kim et al., 2019). These HFD-induced changes in cytokine levels and mitochondrial morphology can be prevented when UCP2 is deleted in microglia. Importantly, deletion of UCP2 in microglia is also associated with more excitatory synaptic inputs onto the POMC neurons, and this leads to increased sensitivity to leptin in POMC neurons and resistance to HFD-induced obesity in mice (Kim et al., 2019). These data suggest that shutting down fatty-acid oxidation and OXPHOS in mitochondria is crucial to reprogram the microglial immunometabolism, reduce cytokine production in reactive microglia, and protect the POMC neurons from HFD-induced dysfunction.

An important issue regarding studies on microglial immunometabolism is that these cells are highly active with their motility and cellular metabolism, logically, none of the *in vitro* experimental approaches can closely mimic *in vivo* conditions (Timmerman et al., 2018). Thus, *in vitro* tools should be only applied when, for example, high cell number is critical for the study (Timmerman et al., 2018).

### Dysregulation of phagocytosis in proinflammatory microglia

TNFα is a major cytokine produced by reactive microglia in obesogenic environments. On one hand, TNFα-activated nuclear factor-kappa B (NF-κB) pathway plays a critical role in the reactive microglia to enhance their pro-inflammatory responses by increased cytokine production and cell proliferation, thus boosting the microglial activity as an autocrine factor (Kuno et al., 2005; Block et al., 2007). On the other hand, TNFα secreted by the reactive microglia acts on neighboring hypothalamic POMC neurons and induces mitochondrial stress (Yi et al., 2017). The TNFα-impaired mitochondrial function might be one of the major causes of HFD-induced POMC neural dysfunction, which is not only observed in the long-term HFD-fed animals (Thaler et al., 2012; Yi et al., 2017; Kim et al., 2019), but also in postmortem hypothalamic tissue of type 2 diabetic patients (Alkemade et al., 2012; Kalsbeek et al., 2020). Correspondingly, restraining the TNFα/NF-κB pathway in microglia can effectively reduce microglial activation and prevent diet-induced obesity (Valdearcos et al., 2017), and blocking the TNFα/NF-κB pathway in POMC neurons can also reduce the diet-induced obesity. This highlights the TNFα/NF-κB pathway in the MBH as a potential brain target for treating obesity.

The reactive microglia in the MBH upon HFD is not only characterized by increased cytokine production, but also impaired phagocytic capacity, as shown by a downregulation of phagocytic indicator cluster of differentiation 68 (CD68) expression during day and night in HFD-fed rats (Milanova et al., 2019). Intriguingly, HFD feeding stimulates microglia to produce more LPL (Gao et al., 2017b). Besides governing lipid uptake for fueling, the LPL-gated phospholipid production is also crucial for phagolysosome formation and turnover (Boulais et al., 2010). Thus, HFD feeding might impair microglial phagocytosis in the MBH, whereas a compensatory mechanism drives more LPL expression and phospholipids production in order to maintain the phagocytic capacity. Not surprisingly, in HFD-fed mice, lacking LPL on microglia, there was a further down-regulation of CD68, and worsened phagocytic capacity, ultimately associated with less POMC-expressing neurons and more vulnerability to HFD-induced metabolic disorders (Gao et al., 2017b). This suggests that lacking a sufficient microglial phagocytosis has a detrimental effect on POMC neural survival upon HFD challenge (Fig. 2).

Consistent with the finds in animal studies, microglial functional changes are also found to be associated with human obesity. This is demonstrated by an increased area covered by microglial cell bodies in the infundibular nucleus (an area equivalents to the ARC in rodents) in obese individuals, accompanied by microglial dystrophy/cytorrhexis (Baufeld et al., 2016). Microglial cytorrhexic dysmorphology are often observed in neurodegenerative diseases, where microglia are over-challenged with immune or phagocytic tasks and eventually become “burn out” (Baron et al., 2014; Streit et al., 2014). Thus, after long-lasting activation, microglial phagocytic inability could occur late in the course of the metabolic disorders, which then eventually contribute to the loss of hypothalamic neurons in the type 2 diabetic patients.

As in HFD-activated microglia, the increased pro-inflammatory cytokines oppose phagocytic capacity, targeting microglial pathway in treating obesity needs to tackle both inflammatory and phagocytic pathways. Hypothalamic cell-specific therapeutic targeting is one of the most promising strategies to create an efficient anti-obesity treatment. The eventually goal is to identify intracellular signaling pathways in the hypothalamic microglia that can be targeted by cell-specific compounds. By normalizing the dysfunctional hypothalamic microglia and the associated innate immune response, as induced by our hypercaloric environment, these compounds should be able to treat obesity and prevent associated metabolic syndromes.

### Hypothalamic astrocytes reactivity in obesity

Similar to microglia, astrocytes also get activated rapidly upon HFD feeding, characterized by increases in the cell number (Thaler et al., 2012; Buckman et al., 2015; Zhang et al., 2017b), and with the NF-κB pathway as an important player in astrogliosis (Buckman et al., 2015; Zhang et al., 2017b). Not surprisingly, the astrocytes-derived cytokine IL-6 (Van Wagoner and Benveniste, 1999) at the downstream of NF-κB pathway (Oeckinghaus et al., 2011) was also found to be elevated within 24 h of HFD exposure (Thaler et al., 2012). Intriguingly, the HFD-stimulated astrogliosis is associated with shortening of the high-order processes of individual astrocyte and impairment of astrocytic process plasticity, mediated by IKKb/NF-kB pathway (Zhang et al., 2017b). It is known that under various pathophysiological conditions, the coating of neurons by astrocytic processes is important for maintaining inter-neuronal communications (Syková, 2004) and rewiring the output of the melanocortin neurocircuit (Horvath et al., 2010). Indeed, the impaired astrocytic process plasticity was associated with dysregulation of extracellular gamma-aminobutyric acid (GABA) (Zhang et al., 2017b), which may affect neurocircuits in different brain regions that control feeding and energy metabolism (Kim et al., 2015; Wu et al., 2015). Two blood-borne metabolic hormones insulin and leptin, with their receptors highly expressed by astrocytes, are found to profoundly regulate astrocytes activity (Kim et al., 2014; García-Cáceres et al., 2016). Deletion of either receptor not only changed astrocytes morphology, but also impacted the activity of the hypothalamic melanocortin circuitry (Kim et al., 2014; García-Cáceres et al., 2016), indicating the essential role of astrocytes in metabolic sensing and regulation in the MBH.

It should be noted that both microglia and astrocytes constitute heterogeneous populations in the brain. The microglial heterogeneity is mainly related to their distinctive molecular markers or morphological diversities (Burns et al., 2020; Masuda et al., 2020; Olah et al., 2020). Moreover, besides the resident microglia, brain also contains macrophages under different pathophysiological conditions (Masuda et al., 2020). Nevertheless, all the microglia and macrophages in the brain share some common markers, such as Cx3cr1, that serve as the promotor for Cre line for genetically manipulation of microglia/macrophages function. Unlike microglia that share common markers, not all astrocytes in the brain can be recognized by a single common marker. The glial fibrillary acidic protein (GFAP) that is commonly used, can only recognized sub-population of astrocytes (Tatsumi et al., 2018). Thus, data obtained by GFAP immunohistology or using GFAP promotor (human or mouse) Cre lines need to be interpreted with caution. Alternatively, a combinational genetic manipulation targeting subpopulation of astrocytes that express 10-formyltetrahydrofolate dehydrogenase (*Aldh1l1*) (Srinivasan et al., 2016) or glutamate aspartate transporter (*Glast*) (Mori et al., 2006), might be essential for the complete understanding of astrocytes function in the context of obesity and metabolic disorders.

## Gut Microbiota and Brain-Regulated Metabolism

### Gut microbiota, brain and metabolism

Microbiota forms an ecological balance with human body during evolution. The symbiotic relationship between gut microbiota and humans provides beneficial effects to human health including: (1) defense against pathogen colonization by nutrient competition and production of antimicrobial substances; (2) fortification of intestinal epithelial barrier and induction of secretory immunoglobulin A synthesis to limit pathogenic bacteria penetration into tissues; (3) facilitation of nutrient absorption by metabolizing indigestible dietary compounds; and (4) participation in the maturation and functionality of the host immune system by providing diverse signals for “tuning” the host immune status (Round and Mazmanian, 2009; Adak and Khan, 2019). Importantly, emerging studies have demonstrated a profound involvement of gut microbiota in an array of diseases through modulating brain neuron function. Given the expanding literature involving the function of gut microbiota, this section will only focus on those that are closely related to brain control of metabolism and obesity.

### The role of gut microbiota in influencing brain-regulated metabolism

Gut microbiota can be influenced by and in turn influence host metabolism. There is a huge change of microbiota community composition in obese and diabetic people (Clemente et al., 2012). Overall, obese people contain low microbiota richness with an increased *Firmicutes* to *Bacteroidetes* ratio, though this conclusion seems controversial in some studies (Patterson et al., 2016). Using metagenomics, researchers found that anti-inflammatory microbial species, like *Faecalibacterium prausnitzii*, are more abundant in lean people with high microbiota richness, while *Bacteroides* and *R*. *gnavus* enrichments are more closely related to obese individuals (Le Chatelier et al., 2013). As to animal models, HFD fed germ-free (GF) mice gain less fat mass compared to mice with normal gut microbiota (Bäckhed et al., 2004), and obese phenotypes can be mimicked on GF mice if they receive fecal microbiota transplantation (FMT) from obese mice (Turnbaugh et al., 2006).

**Figure 3:**
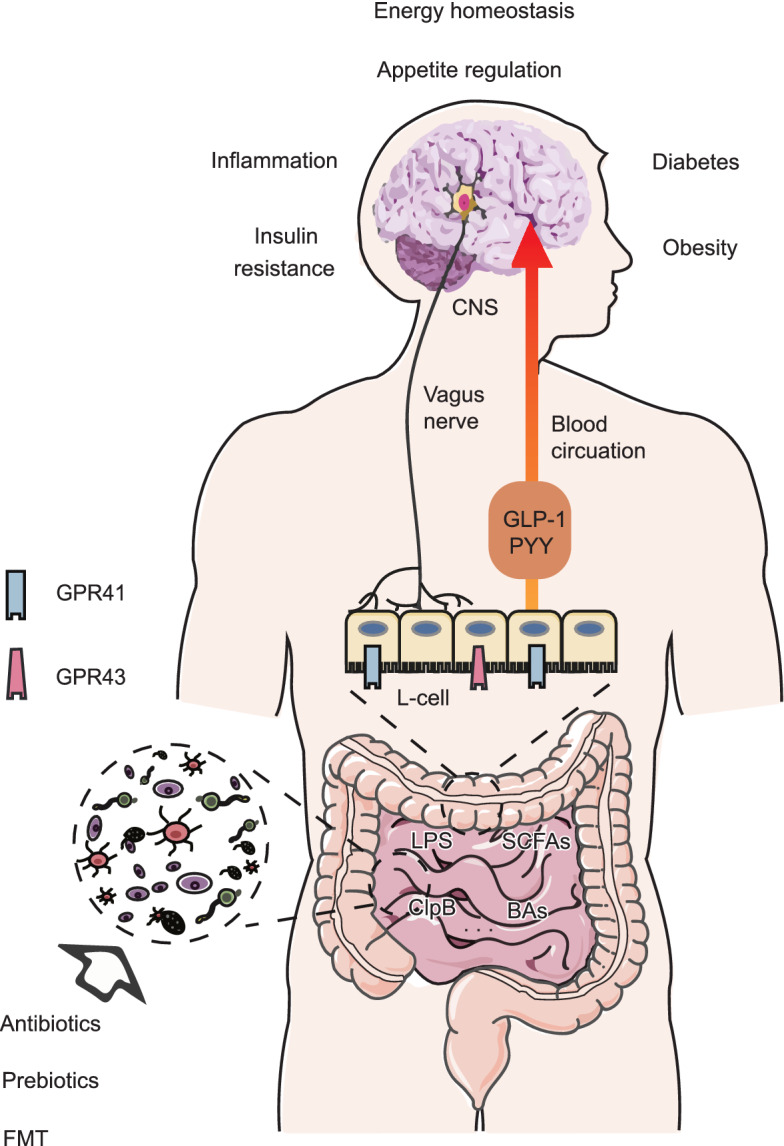
**Gut microbiota involved in a complex interaction with host metabolism.** The gut microbiota influences neural control of metabolism through multiple ways. SCFAs produced by gut microbiota can activate GPR41 and GPR43 receptors on intestine L cells. After being activated, L cells produce GLP-1 and PYY peptides, which act in the brain by either blood circulation or vagus nerve to reduce food intake and balance glucose metabolism. BAs are another kind of microbiota metabolites that promote GLP-1 production from intestine L cells. ClpB is the only known bacterial protein that can reduce food intake by gut-brain axis. HFD intake causes increased Gram-negative bacteria proportion, LPS concentration and systemic inflammation. As LPS can activate vagus nerve, gut microbiota may modulate systemic and brain inflammatory levels by LPS production. To be noted, when studying functional roles of specific microbiota clusters, antibodies/prebiotics supplements and FMT are the most common approaches to choose

The “gut-brain axis” concept describes a bidirectional interaction between the brain and gut. The “microbiota-gut-brain axis” is subsequently developed as gut microbiota is significantly involved in the gut-brain axis (Cryan et al., 2019). Now, studies have found that gut microbiota can produce metabolites and proteins to modulate neuron-regulated host metabolism, although the exact mechanisms are largely unknown. Here, we discuss current progress in exploring the role of gut microbiota in neural control of metabolism (Fig. 3), and raise unsolved questions for future directions (For techniques involved in microbiota researches, please see review Cryan et al., 2019 for details).

Gut microbiota produce short-chain fatty acid (SCFA) by fermentation of organic compounds such as carbohydrates, lipids and dietary fibers. SCFAs influence gut-brain axis mostly based on endocrine-circulating system. Apart from crossing BBB directly (Mitchell et al., 2011), SCFAs are endogenous ligands of G-protein coupled receptor 41 and 43 (GPR41 and GPR43). Intestine L cells express these two receptors as well as glucagon-like peptide-1 (GLP-1) and peptide YY (PYY) (Karaki et al., 2006), so it is likely that some SCFAs promote GLP-1 and PYY production by binding to GPR41 and GPR43. Indeed, degeneration of dietary fibers by microbiota increased GLP-1 (Cani et al., 2004) and PYY (Delzenne et al., 2005) levels and modulate food intake by acting in the brain (Murphy and Bloom, 2006). Consistently, the feeding-suppressing effects of SCFAs require the intact vagal neurons, where GLP-1 and PYY are known to act (Baraboi et al., 2010; Krieger et al., 2015, 2016). For example, hepatic vagotomy or capsaicin treatment (which denervates vagal neurons) attenuates or abolishes effects of intraperitoneal (IP) injection of acetate, propionate and butyrate to decrease food intake (C et al., 2018). In addition, propionate supplement from diet also induces dramatic activation of the dorsal vagal complex (DVC), which can be reduced by capsaicin (De Vadder et al., 2014). Further, subdiaphragmatic vagotomy blunts food-suppression effect of butyrate (Li et al., 2018b), supporting that SCFAs act, at least in part, through vagal afferent neurons.

Bile acids (BAs) are another kind of metabolites associated with microbiota. BAs can not only activate intestine L cells for GLP-1 production (Brighton et al., 2015), but also induce fibroblast growth factor 19 (FGF19) production from enterocytes (Steven et al., 2015). GLP-1, FGF19 and BAs themselves can all cross the BBB to modulate feeding and glucose metabolism by binding to respective receptors on neurons (Jameson et al., 2020).

In addition to the microbiota-associated metabolites, bacterial proteins may also influence metabolism via the gut-brain axis. For example, the bacterial protein caseinolytic protease B (ClpB) was shown to mimic α-MSH and act in the brain to reduce food intake (Breton et al., 2016).

Further, gut microbiota themselves can influence brain function. For example, *Lactobacillus rhamnosus* supplement increases GABA levels in the brain and reduces anxiety controlled by the vagus nerve (Bravo et al., 2011), although it remains unclear whether such mechanisms also influence brain functions that are relevant to metabolic control.

Notably, obesity in HFD-fed animals is associated with increased Gram-negative bacteria proportion in gut microbiota, with subsequent increased lipopolysaccharide (LPS) plasma concentration, systemic inflammation and obesity (Cani et al., 2008). Since LSP can activate vagal afferent neurons (Klingbeil and de La Serre, 2018), such association suggest that dysregulated gut microbiota may contribute to diet-induced obesity, although this cause–effect relation remains to be definitively tested (Dalby et al., 2018).

In summary, current researches in the connection between gut microbiota, brain and metabolism have suggested that compositions of gut microbiota changes in association with BMI. Gut microbiota regulates neural control of metabolism, probably via endocrine system and vagus nerve.

## Stress, Feeding and Obesity Development

### Stress, anxiety and related emotional modalities

The mental and physiological processes involved in stress and related emotional modalities are complex and often difficult to precisely define. In general, stress is described as a state of mental or emotional strain or tension resulting from adverse or very demanding circumstances. Anxiety is the reaction to situations perceived as stressful and dangerous. Both stress and anxiety are a normal part of life and elicits changes with physical, mental, and emotional responses necessary for maintaining welfare and survival of individuals and species. However, a disproportional level of anxiety with excessive nervousness and fear without apparent triggers leads to anxiety disorders (Patriquin and Mathew, 2017). Depression is normally regarded as a generalized form of anxiety with symptoms of anhedonia and reduced motivation, a major form of psychiatric disorders and can result from chronic experience of stress and anxiety (Kessler, 1997; Pizzagalli, 2014). Despite potential difference in stress and related emotional modalities between humans and rodents (Tye, 2018), studies in rodent models on human-emotion like behaviors have provided important insights on neurobiology related to stress related behaviors. Given the vast literature on the topic of stress and related behaviors, this review only focuses on stress related to feeding and obesity development.

### Stress and body weight regulation

Corticotropin releasing hormone (CRH) neurons, widely distributed in the brain, are thought to be one of the first responders in orchestrating behavioral and hormonal responses to stress (Deussing and Chen, 2018). Hypothalamic CRH neurons, a major subset of brain CRH neurons, are located in the PVH, a key brain region for feeding and body weight regulation, and therefore likely serve as a link between metabolism and stress responses. These neurons are known to elicit neuroendocrine responses through the autonomic nervous system and hypothalamus–pituitary–adrenal axis (HPA). Extensive studies in humans implicate the HPA axis in obesity development. Uncontrolled glucocorticoid levels are associated with hyperphagia, insulin resistance, obesity, and diabetes, as observed in human Cushing’s diseases (Pivonello et al., 2015). Heightened glucocorticoid levels have been suggested to increase feeding and obesity through insulin action and producing a bias toward increased motivation to eat and decreased executive function through altering brain cognitive function (Sinha and Jastreboff, 2013). Consistent with the observations in humans, a heightened HPA axis is also associated with obese animal models including leptin deficient *ob*/*ob* mouse models (Huang et al., 1998). Interestingly, manipulation of CRH expression in adult mice affects diet preference (Okamoto et al., 2018).

Given the consequence of chronic stress on emotional changes including anxiety and depression (Berry et al., 2012), it is not surprising that there is a strong association between psychiatric disorders including anxiety and depression, and obesity in humans (Siervo et al., 2009). It has been proposed that stress-induced emotional eating links anxiety/depression and obesity through alterations in cortisol and brain reward circuitry, leading to promotion of compulsive overeating (Dallman, 2010; Nederkoorn et al., 2015). Conversely, eating disorders are often accompanied with alterations in emotion including stress, anxiety and aggression (Sweeney and Yang, 2017). Importantly, treatments of psychological disorders are known to be associated with obesity development (Domecq et al., 2015). Collectively, these observations argue that chronic stress and consequent emotional derangements including anxiety and depression contribute to human obesity.

Given the substantial evidence in humans on chronic stress and obesity, it is surprising that results using animal stress models are inconsistent (Razzoli and Bartolomucci, 2016). Some studies conclude that stress reduces feeding and body weight (Michel et al., 2005; Liu et al., 2014; Harris, 2017; Qu et al., 2020), while others show the opposite that stress induces weight gain (Lutter et al., 2008; Melhorn et al., 2010; Razzoli et al., 2015). One important reason, among others, may lie in the nature of stressors imposed (Harris, 2015; Razzoli et al., 2017). Self-reported stress in humans, as normally conducted in human studies, is largely chronic and psychological in nature, likely resulting from socioeconomic pressure. Thus, it is rather difficult to mimic the same chronic stress situation in rodents. Those reports on chronic stress reducing body weight in rodents often use repeated environmental stresses, i.e., foot shock or restraint (Michel et al., 2005; Liu et al., 2014; Harris, 2017), which are essentially repeated acute stressors with periods of relieving phases. Acute stress is known to reduce feeding in short term (Maniscalco and Rinaman, 2017; Terrill et al., 2018), and acute brain action of CRH and its related urocortins reduces feeding (Stengel and Tache, 2014). In contrast, chronic stress in animal models with weight gain is normally induced by psychological social defeat (Lutter et al., 2008; Melhorn et al., 2010; Razzoli et al., 2015). Chronic psychological stress produces social avoidance and other signs common to anxiety and depression patients (Chiba et al., 2012; Venzala et al., 2013), and therefore may better mimic chronic stress in humans. Importantly, human genetic studies are beginning to identify candidate genes underlying the association between stress/anxiety and obesity, which can be confirmed in mice (Meier et al., 2019), supporting that the effect of chronic stress on obesity may be evolutionally conserved. A combination of mouse genetics and chronic psychological stress models may help to identify key brain neurons and pathways in mediating the effect of chronic stress on obesity.

### HFD, stress and obesity

As human obesity is contributed by both HFD and stress, it is imperative to consider whether HFD and chronic stress interact to precipitate obesity development. HFD alters both basal and stress-induced HPA axis, reduces corticosterone rhythmicity (Auvinen et al., 2012; Kalyani et al., 2016), induces anxiety-like behaviors (Gomes et al., 2020), and suppresses stress responses (Appiakannan et al., 2019). Conversely, stress aggravates HFD-induced insulin resistance and heightened glucocorticoid action worsens obesity and hyperglycemia induced by HFD (Bartolomucci et al., 2009; Tsai et al., 2018; Garcia-Eguren et al., 2019). These observations strongly suggest that HFD and chronic stress synergistically promote obesity and associated metabolic syndrome.

Given the similar effect of HFD and chronic stress on obesity development, it is important to identify the underlying neural basis. Studies focusing on the PVH CRH neurons have suggested an importance of the responsiveness of these neurons in HFD-induced obesity. PVH CRH neurons exhibit a diurnal pattern of activity, which orchestrates a diurnal pattern of the HPA activity. HFD alters synaptic inputs to PVH neurons (Mazier et al., 2019), and blunts diurnal patterns of CRH expression (Auvinen et al., 2012), and therefore may alter the activity pattern of these neurons. Importantly, clamping the activity level of PVH CRH neurons either at high or low levels, which render these neurons not responsive to either stressors or nutritional challenges, mimicking HFD effects on obesity development (Zhu et al., 2020), which illustrates a potential importance of dynamic responses of PVH CRH neurons to environmental or nutritional challenges in HFD-induced obesity. Interestingly, HFD reduces responsiveness of CRH and AgRP neurons (Beutler et al., 2020; Zhu et al., 2020). It is conceivable that the responsiveness of neurons underlies their activity pattern by integrating dynamic changes in synaptic and hormonal inputs, and therefore shapes the diurnal pattern activity of these neurons (Ulrich-Lai and Herman, 2009). In addition, intrinsic neuron properties, for example, the core clock genes are known to drive an intricate gene network that ultimately orchestrates neuron responsiveness and activity patterns related to stress (Hood and Amir, 2018). Deletion of brain and muscle arnt-like 1 (*Bmal1*), a key core clock gene, in the PVH reduces the responsiveness of these neurons to external stressful stimuli, which blunts diurnal rhythms in energy expenditure and feeding, and causes obesity (Kim et al., 2020). HFD is known to disrupt diurnal rhythms in feeding and metabolism (Hatori et al., 2012). Thus, studies aiming to examine whether neuron responsiveness underlying the interplay between stress/HFD and circadian rhythms will provide important insights on the role of stress in obesity development.

### Common neural pathways in feeding and stress-related emotional modalities

Emerging studies suggest that feeding reciprocally affects emotion. AgRP neurons in the ARC, a key group of feeding-promoting neurons, are inhibited by stress stimuli while their activation promotes feeding and reduces behavioral signs of anxiety (Dietrich et al., 2015; Burnett et al., 2016; Padilla et al., 2016). GABAergic lateral hypothalamic area (LHA) neurons, while potently promoting feeding, produce place preference and rewarding effects and increases aggression and predatory behaviors (Jennings et al., 2015; Nieh et al., 2015; Li et al., 2018a; Mangieri et al., 2018; Cassidy et al., 2019), behavioral signs of reduced anxiety and stress perception. In contrast, glutamatergic LHA neurons reduce feeding through promoting behavior signs of stress and anxiety (Nieh et al., 2015; Stamatakis et al., 2016; Mangieri et al., 2018). PVH neurons, in addition to the well-studied CRH neurons, contain other types of neurons that send projections to the lateral septum (LS), a known brain region involved in aggression and anxiety, to inhibit feeding while promoting anxiety-like behaviors and reducing aggression (Xu et al., 2019). In particular, double knockout of synapse-associated protein 90/postsynaptic density-95-associated protein-3 (*Sapap3*), a key gene involved in anxiety-like behaviors, and *Mc4r*, one of the most frequent obesogenic genes in humans, completely rescues anxiety-like behaviors seen in *Sapap3* knockouts and obesity in *Mc4r* knockouts (Xu et al., 2013), demonstrating an intimate interaction between the two seemingly unrelated genes. Thus, these and other findings from rodents argue that feeding and stress-related emotional modalities are regulated by a shared neuronal network widely distributed in the brain (Sweeney and Yang, 2017). Consistently, studies in humans suggest a profound effect of emotional feeding on obesity development, and aversive compulsivity and refusal to feed are co-existent in anorexia nervosa (Mattar et al., 2012; Godier and Park, 2014), suggesting that the co-regulation of feeding and stress-related emotional modalities is evolutionarily conserved. Thus, it is of importance to examine how these divergent brain regions respond to stress and HFD, and contribute to the current obesity epidemic.

## Aging in the Central Nervous System of Organisms

### Neuroendocrinal control of *C. elegans* aging from metabolic regulation

*Caenorhabditis elegans* (*C*. *elegans*) model was first introduced as a genetic model organism more than 50 years ago. In laboratory environment, the lifespan of *C*. *elegans* is approximately 3 weeks, and this nematode can also develop to a fertile adult in 3 days. Longevity studies using *C*. *elegans* have been conducted for decades, and among others, insulin/insulin-like growth factor-1 signaling (IIS) pathway has been best appreciated for the influence on longevity of this animal (Morris et al., 1996). There are three main components of the IIS pathway in *C. elegans*, including DAF-2 (*C. elegans* homolog of the insulin/insulin-like growth), AGE-1 (*C. elegans* homolog of phosphatidylinositol 3-kinase), and DAF-16 (*C*. *elegans* homolog of the forkhead box FoxO transcription factor). The mutants that regulate the activity of these signaling components have significant effects on increasing the lifespan of *C*. *elegans* (Friedman and Johnson, 1988a, b; Kenyon et al., 1993). The IIS pathway is also important for sensory perception involved in the regulation of the lifespan of *C*. *elegans*. While chemosensation-deficient *C*. *elegans* increased lifespan, chemosensory defects or elimination of gustatory neurons can dramatically decrease the lifespan effect of *daf-16* mutation (Apfeld and Kenyon, 1999; Alcedo and Kenyon, 2004). The underlying mechanisms could be partially related to increased abilities of worms to handle various environmental and physiological stresses. In the long-lived strain of *C*. *elegans*, it was found that the increased stress resistance with reduced insulin signaling (Kenyon, 2010). Consistently, lifespan studies of *C*. *elegans* with genetic manipulation increasing stress resistance suggest that stress response is an important mechanism for modulating the lifespan (Durieux et al., 2011; Taylor and Dillin, 2013). Studies further showed that blocking of chemosensation can increase the nuclear translocation of DAF-16 in *C*. *elegans*, and decrease in DAF-16 nuclear localization can be induced through re-exposure to food in food-deprived *C*. *elegans* (Artan et al., 2016). DAF-9/P450, which is responsible for lipid hormone signaling in *C*. *elegans*, has also been shown to regulate larval development and aging (Gerisch et al., 2001; Jia et al., 2002; Rottiers et al., 2006). Temperature is one of the important factors in controlling lifespan by altering the metabolic rates (Jeong et al., 2012; Xiao et al., 2015). Ablation of thermosensory neurons can decrease the lifespan of *C*. *elegans* at 25 °C (Lee and Kenyon, 2009). Transient receptor potential cation channel subfamily V member 1 (TRPV1), a transient receptor potential (TRP) cation channel, is one of the major pain receptors that respond to noxious heat in vertebrates. Previous study showed that TRPV1-null mice increased lifespan compared to wild type (WT). Similarly, TRPV double-null mutant worms with deletion of both *osm-9* and *ocr-2* can prolong their lifespan (Riera et al., 2014). *C*. *elegans* uses three receptors, GCY-8, GCY-18 and GCY-23 as thermal sensors, and while triple mutant of these receptors did not detect an increasing temperature, it can lead to a shortened lifespan of worms at 25 °C (Inada et al., 2006; Chen et al., 2016). The response of organisms to various environmental and physiological stresses can determine their lifespan. In the long-lived strain of *C*. *elegans*, it was found that the increased stress resistance with reduced insulin signaling (Kenyon, 2010). In addition, lifespan studies of *C*. *elegans* with genetic manipulation increasing stress resistance suggest that the stress response pathway is an important mechanism for regulating lifespan (Durieux et al., 2011; Taylor and Dillin, 2013).

### Hypothalamic control of aging in mammals: an emerging and exciting theme

One of the characteristics of physiological changes that occur during aging is the change in energy homeostasis (Hildrum et al., 2007). Despite being a healthy old man without degenerative disease, changes in metabolic activity can include sarcopenic obesity, a problem of fat gain but muscle loss in body composition. The loss of appetite in the elderly also leads to an imbalance between energy metabolism and nutrition (Wolden-Hanson et al., 2004). The ARC, located in the bottom of the hypothalamus, play important roles in regulating food uptake to maintain energy homeostasis. The ARC consists of orexigenic AgRP/neuropeptide Y (NPY) neurons and anorexigenic POMC/cocaine- and amphetamine-regulated transcript (CART) neurons, and notably POMC activity in aged mice was significantly reduced (Yang et al., 2012), but *Pomc* mRNA levels in ARC were not changed in young and middle-aged mice (Burke et al., 2014). Age-dependent metabolic alteration in aged rats was alleviated by overexpressing POMC gene in the hypothalamic ARC, including improved glucose metabolism, insulin sensitivity and reduced food consumption in aged rats (Li et al., 2005). NPY protein and NPY receptor levels also decrease in hypothalamic area of aged rats (Kowalski et al., 1992; Veyrat-Durebex et al., 2013). Through studies during the recent decade, it was discovered that the hypothalamus plays causal roles in systematic aging (Satoh et al., 2013; Zhang et al., 2013, 2017a). Previously, hypothalamic atrophy and signaling defects have been found in aging-related neurodegenerative diseases (Loskutova et al., 2010; Ishii and Iadecola, 2015; Vercruysse et al., 2018). The concept of NF-κB microinflammation in aging is clearly in alignment with its general role in metabolic syndrome which has been previously appreciated (Cai and Khor, 2019). Through experimental models, mid-aged rodents with an increase of hypothalamic NF-κB activity were reported to present aging acceleration and lifespan loss, while inhibition of NF-κB signaling in the hypothalamus was sufficient to lead to aging slowdown and a lifespan increase (Zhang et al., 2013). Caloric restriction, an effective dietary approach for aging intervention, has been shown to reduce hypothalamic gliosis and inflammatory changes (Yan et al., 2014; Sadagurski et al., 2017). Also, it should be mentioned that anti-aging drugs such as acarbose (Kothari et al., 2016), aspirin (Ong et al., 2007), rapamycin (Thomson et al., 2009), and metformin (Barzilai et al., 2016) all seem to be counter inflammatory. Thus, future research is warranted to investigate if hypothalamic inflammation is a common mechanism shared by these medical and non-medical anti-aging approaches.

### Hypothalamic control of aging: targeting hypothalamic stem cells

In aging brains, one of the physiological features is a decreased production of new functional neurons from adult neural stem cells (Seib and Martin-Villalba, 2015). The derogatory effects of NF-κB signaling in the hypothalamus on aging seem to be causally related to its inhibition on gonadotropin-stimulating hormone (GnRH), a hypothalamic peptide which is classically known for its regulation on reproductive physiology but was found to important for promoting neurogenesis in this study (Zhang et al., 2013). Previous studies have found that hypothalamic neural stem/progenitor cells (htNSCs) are present in adult mice and physiologically important for hypothalamic long-term regulation over metabolic balance (Li et al., 2012). In 2017, it was discovered that htNSCs can function to supervise the process of aging while age-dependent loss of these cells is responsible for aging acceleration, and the mechanism is critically related to a novel endocrine function of these cells in secreting miRNA exosomes (Zhang et al., 2017a). This finding was consistent with the appreciation that decreased neurogenesis correlates with aging and the development of aging-related disorders (Greenberg and Jin, 2006; Molofsky et al., 2006; Encinas et al., 2011; Villeda et al., 2011; Sun et al., 2013; Baruch et al., 2014). As htNSC transplantation was tested for anti-aging effects, poor survival of implanted htNSCs was directly correlated with inflammatory status, but NF-κB inhibition in htNSCs was required for their survival (Li et al., 2012; Zhang et al., 2017a). A recent study have revealed a mechanism that seems important for aging-associated loss of htNSCs due to a noncoding long RNA named *Hnscr* (Xiao et al., 2020). In this study, it was reported that specific depletion of *Hnscr* in htNSCs accelerated the senescence of htNSCs and promoted aging-related physiological symptoms. The underlying molecular basis is related to the function of *Hnscr* in stabilizing Y-box binding protein 1 (YB-1) which is important for anti-apoptosis. Overall, the role of hypothalamic stem cells in systemic aging is not only consistent with neurodegenerative paradigm of aging and but has added a new perspective of aging mechanism from neuroendocrine regulation and dysregulation.

## The Next Decade of Neural Control of Metabolism and Aging

It has become increasingly clear that human obesity is driven by both genetics and environmental changes. Research in last decades, especially recent emerging techniques including sophisticated mouse genetics, optogenetic manipulation, calcium indicator and single-cell sequencing have revealed ever-growing brain sites and neurons that are implicated in feeding and likely body weight regulation. However, it remains to be established which of these brain regions are relevant to human obesity development and therefore can be used as therapeutic targets against human obesity. This is thus critical to identify dysfunctions in brain neurons and pathways that underlie human obesity. Given the high palatable food environment as a strong driver for the current obesity epidemic, special emphasis should be put on the action of HFD on brain neurons. Recent studies have suggested a defective neuron responsiveness in AgRP neurons and CRH neurons by HFD (Beutler et al., 2020; Mazzone et al., 2020; Zhu et al., 2020), which may play a critical role in diet-induced obesity. Here we briefly speculate on a few major directions that we think are critical to unravel key brain neurons and neural pathways that are most relevant to human obesity. It is important to point out that these directions may represent a few of many potential important ones on obesity research. To go further in this field, several directions would be expected as below:

### Single-cell sequencing

Single-cell sequencing is now a useful tool precisely combining neuron sub-types to metabolic functions. However, much efforts should be paid before completing single-cell sequencing. Subtle division of neuronal subtypes and brain regions are required for appropriate sample sites. Different sequencing methods (e.g., single-cell sequencing and translating ribosome affinity purification sequencing) should be combined to map the exact function-related neuron population. If possible, improved cell immunofluorescence with computational remodeling could provide anatomical evidence in parallel. After neuronal subdivision, how to label and manipulate those neurons is still a question to be solved.

### Human relevance

Through decades of research using cells and/or experimental animals as model systems, a great deal of knowledge has been obtained regarding the molecular, cellular and systemic mechanisms for the regulation of whole-body metabolism in the studied models. However, a major question in the field still remains unsolved: what cause metabolic disorders in humans? Since most of the obesity-associated human variants affect genes that are enriched in the brain (Locke et al., 2015), we suggest to bring together the diverse expertise in human metabolic research and basic neuroendocrinology, to reveal the fundamental genetic basis and the neuroendocrine mechanisms that regulate metabolic homeostasis not only in animals but also in humans.

### Sex difference

While the sex difference in energy homeostasis and other physiological systems are being increasingly recognized (Arnold, 2014), the underlying mechanisms remain elusive. In addition to the genes encoded by the sex chromosomes and the gonadal hormones (and their receptors), we predict that a large number of autosome-encoded genes also contribute to the sex differences in energy balance. However, a lot of such genes have been missed simply because female animals are purposely omitted in most studies to reduce costs and efforts. Therefore we suggest that all pharmacological and genetic investigations into energy balance in animal models should include both males and females, as required by the funding agencies, e.g., the National Institutes of Health (NIH) (Tannenbaum et al., 2016).

### Immunometabolism

The impact of immune system on brain-regulated metabolism should be emphasized in future. As to the brain, the role of microglia and astrocytes in regulating metabolic disorder has long been ignored. With bulk of data accumulated in the recent years, the essential roles played by these glial cells become clearer than ever. Yet, whether and how microglia in the hypothalamus precisely detect diet-induced metabolic stresses, how microglia interact with astrocytes, and to which extent the heterogeneity is when different hypothalamic neurons interact with different glial cells are remain to be investigated to demonstrate this multi-system collaboration. Nevertheless, as we have discussed above, the ultimate role of glial population is to serves as the “supporting unit” for neurons to function, it is plausibly to prospect that glial cells are readily to adapt their immune and metabolic functions to match the demands of neuroprotection in the context of metabolic stress.

### Gut microbiota

It is necessary to comprehend CNS-peripheral tissue network. We have known that gut microbiota produces different compounds and participates in brain control of metabolism. However, many details remain to be explored.

First, this field lack comprehensive understandings from microbiota to brain. Compared with compounds gut microbiota produces, evidence of how microbiota responds to environment, produces those compounds and finally mediate CNS is elusive. Second, the role of vagus nerve in innervating gut microbiota and brain-regulated metabolism need more clarification in future. Finally, further studies should consider human-subject reproduction, establishing causal link of microbiota and brain-metabolic diseases, and finally promote related therapeutic methods.

### Stress

It has become increasingly clear from extensive human behavioral studies that both chronic stress and HFD contribute to the current obesity epidemic. In contrast to HFD, the mechanism underlying the effect of stress on body weight has received much less attention in rodent research. One major reason is that it is difficult to model chronic stress-induced emotional modalities in rodents. Identification of a reliable chronic stress model that recapitulates the effect of human chronic stress is a critical next step to unravel the underlying mechanism for chronic stress induced obesity in humans. It appears that chronic social stress shows some promises in mimicking the effect on feeding and body weight of human chronic stress. Given the availability of imaging data from humans with chronic stress, future studies using rodent brain imaging may help reveal whether there are similar changes in brain imaging. Given the complex nature of underlying neurocircuitry for both feeding and stress-related emotional modalities (Yau and Potenza, 2013; Herzog, 2020), many brain neurons and neural pathways including traditionally viewed as feeding and emotion brain centers are likely to be involved. Emerging observations demonstrate that feeding brain centers are deeply involved in stress-related emotional modalities and emotional brain centers inherently regulate feeding behaviors. The cellular mechanism underlying the effect of HFD and chronic stress is largely unknown. Notably, HFD blunts PVH CRH neuron responsiveness and the diurnal rhythms of downstream HPA axis, suggesting disruptive neuron responsiveness as a potential cellular mechanism. It is critical to examine whether chronic stress also induce a similar effect on these neurons. It is clear that many brain neurons and neural pathways are involved in chronic stress responses, feeding and body weight, it is thus imperative to understand the relative contribution of these brain regions and pathways in mediating chronic stress and HFD on obesity development, especially those brain regions that mediate the synergistic effect of HFD and chronic stress on weight gain.

### Aging

Recent research has led to appreciation the hypothalamic basis for developing metabolic syndrome and aging involving some overlapping cellular and signaling pathways through NF-κB-dependent microinflammatory induction in neurons, glial cells and hypothalamic neural stem/progenitor cells. With these findings which have begun to shed a light on this important topic, there are many remarkable questions which can be asked, and the following is to briefly list a few perspectives for researchers to consider for future studies.

**Figure 4:**
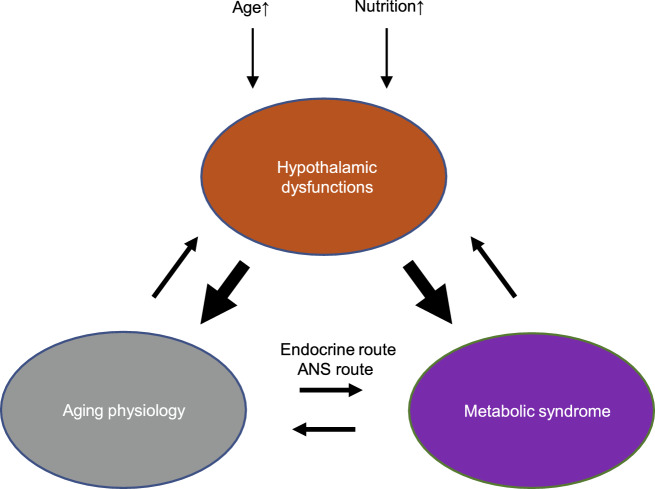
**Hypothalamic scheme for connecting aging with metabolic disorders**. The diagram depicts the backbone relationships among the hypothalamus, aging and metabolic syndrome, suggesting that hypothalamic dysfunctions represent a key cause for initiating the onset of aging and metabolic syndromes although also individually depending on the context of age and chronic nutritional status but with some overlapping processes through the changes in neuroendocrine system and autonomic nervous system (ANS) which are important for hypothalamic and central control of whole-body physiological balance and homeostasis. Over a chronic term, aging and metabolic syndrome physiological changes can not only enhance each other but also reinforce hypothalamic dysfunctions, thus keeping the mechanisms and outcomes in a self-sustaining loop

First of all, given the close relationships among hypothalamic dysfunction, aging and metabolic syndrome (Fig. 4), it is necessary to deeply understand how these various aspects of general pathological and pathophysiological domains are biologically and programmatically integrated. Secondly, regarding the underlying molecular operation, given the critical role of hypothalamic NF-κB as revealed in a good body of literature, it will be informative to investigate how NF-κB might interact with other aging-regulating molecular events such as SIRTs, Adenosine 5′-monophosphate (AMP)-activated protein kinase (AMPK) and mammalian target of rapamycin (mTOR) signaling and if there are NF-κB-unrelated molecular pathways to be critically accountable. Thirdly, while animal models have revealed the causal contribution of hypothalamic microinflammation to the hypothalamic mechanism of aging and related metabolic syndrome, it should be highly valuable to profile if these inflammatory changes occur in human conditions and address the importance of this hypothalamic microinflammatory condition in terms of human aging and related disease, Finally, as there has been a great deal of interest to develop anti-inflammatory therapeutical approaches, current clinical anti-inflammatory options have limited features and are more suitable for solving classical inflammation due to conditions such as infection rather than non-classical microinflammation in aging and related disorders. Thus, new anti-inflammatory approaches should be explored, for example, hypothalamic stem cell exosome-based intervention, which could be more effective in targeting aging-related microinflammation and neuroinflammation. Besides, to incorporate aging-related genes and neural pathways from animal studies into applications will be worthy of future clinical research.

## Abbreviations

5-HT, 5-hydroxytryptamine; 5-HT_2C_R, 5-hydroxytryptamine 2C receptor; ADCY3, adenylate cyclase 3; AGEs, advanced glycation end-products; AgRP, agouti-related peptide; ALDH1L1, 10-formyltetrahydrofolate dehydrogenase; AMP, adenosine 5‘-monophosphate; AMPK, adenosine 5′-monophosphate-activated protein kinase; ANKRD26, ankyrin repeat domain 26; ANS, autonomic nervous system, APOE, apolipoprotein E; AR, androgen receptor; ARC, arcuate nucleus of hypothalamus; ARNT2, Aryl hydrocarbon receptor nuclear translocator 2; AT2R, angiotensin II receptor; BAs, bile acids; BBB, blood-brain barrier; BDNF, brain-derived neurotropic factor; BMAL1, brain and muscle arnt-like 1; BMI, body mass index; *C*. *elegans*, *Caenorhabditis elegans*; CART, cocaine- and amphetamine-regulated transcript; CD68, cluster of differentiation 68; CEP19, centrosomal protein 19; ClpB, caseinolytic protease B; CNS, central nervous system; CRH, corticotropin releasing hormone; CRISPR, clustered regularly interspaced short palindromic repeats; CX3CL1, C-X3-C motif chemokine ligand 1; dLS, dorsal lateral septal nuclei; DMH, dorsomedial hypothalamus; DRN, dorsal raphe nucleus; DVC, dorsal vagal complex; ENU, N-ethyl-N-nitrosourea; ERα, estrogen receptor alpha (also called estrogen receptor 1, ESR1); ERβ, estrogen receptor beta; FGF19, fibroblast growth factor 19; FMT, fecal microbiota transplantation; FTO, FTO alpha-ketoglutarate dependent dioxygenase in *Homo sapiens* and fat mass and obesity associated in *Mus musculus*; GABA, gamma-aminobutyric acid; GF, germ-free; GLAST, glutamate aspartate transporter; GLP-1, glucagon-like peptide-1; Glp1R, glucagon-like peptide-1 receptor; GnRH, gonadotropin-stimulating hormone; GPR30, G protein-coupled receptor 30; GPR41, G protein-coupled receptor 41; GPR43, G protein-coupled receptor 43; GPR54, G protein-coupled receptor 54; GWAS, genome-wide association studies; HFD, high-fat diet; HK2, hexokinase 2; HPA, hypothalamus-pituitary-adrenal axis; htNSCs, hypothalamic neural stem/progenitor cells; IIS, insulin/insulin-like growth factor-1 signaling; IL-1β, interleukin 1 beta; IL-6, interleukin- 6;IP, intraperitoneal; Kiss1, kisspeptin; LEPR, leptin receptor; LHA, lateral hypothalamic area; LPL, lipoprotein lipase; LPS, lipopolysaccharide; LS, lateral septum; MBH, mediobasal hypothalamus; MC4R, melanocortin 4 receptor; ME, median eminence; MeA, medial amygdala; mTOR, mammalian target of rapamycin; MYT1L, myelin transcription factor 1 like; NCOA1, nuclear receptor coactivator 1; NF-κB, nuclear factor-kappa B; NIH, National Institutes of Health; NPY, neuropeptide Y; NREM, non-rapid eye movement; nsSNP, nonsynonymous single‐nucleotide polymorphisms; NTRK2, neurotrophic receptor tyrosine kinase 2; NTS, nucleus of the solitary tract; OGT, O-GlcNAc transferase; OVX, ovariectomy; OXPHOS, oxidative phosphorylation; Oxtr, oxytocin receptor; POA, preoptic area; POMC, pro-opiomelanocortin; PVH, paraventricular nucleus of the hypothalamus; PYY, peptide YY; ROS, oxygen species; Rprm, reprimo; Sapap3, synapse-associated protein 90/postsynaptic density-95-associated protein-3; SCFA, short-chain fatty acid; SCN, suprachiasmatic nucleus; SH2B1, SH2B adaptor protein 1; SIM1, single-minded 1; SIRT1, Sirtuin 1; SNP, single nucleotide polymorphism; SNV, single nucleotide variants; SRC-1, steroid receptor co-activator-1; SRY, sex determining region Y; SST, somatostatin; STAT3, signal transducer and activator of transcription 3; Tac1, tachykinin 1; TLR4. Toll-like receptor-4; TNFα, tumor necrosis factor alpha; TrkB, tropomyosin-related kinase B; TRP, transient receptor potential; TrpC5, transient receptor potential channel 5; TRPV1, transient receptor potential cation channel subfamily V member 1; UCP2, uncoupling protein 2; Vglut2, vesicular glutamate transporter2; VMH, ventromedial hypothalamus; WT, wide type; YB-1, Y-box binding protein 1; α-MSH, alpha-melanocyte-stimulating hormone; β-MSH, beta-melanocyte-stimulating hormone
